# Calcineurin activation influences muscle phenotype in a muscle-specific fashion

**DOI:** 10.1186/1471-2121-5-28

**Published:** 2004-07-28

**Authors:** Robert J Talmadge, Jeffrey S Otis, Matthew R Rittler, Nicole D Garcia, Shelly R Spencer, Simon J Lees, Francisco J Naya

**Affiliations:** 1 Department of Biological Sciences, California State Polytechnic University, Pomona, CA 91768, USA; 2 Department of Human Nutrition, Foods and Exercise, Virginia Polytechnic Institute and State University, Blacksburg, VA 24061, USA; 3 Department of Molecular Biology, University of Texas Southwestern Medical Center, Dallas, TX 75390, USA

## Abstract

**Background:**

The calcium activated protein phosphatase 2B, also known as calcineurin, has been implicated as a cell signaling molecule involved with transduction of physiological signals (free cytosolic Ca^2+^) into molecular signals that influence the expression of phenotype-specific genes in skeletal muscle. In the present study we address the role of calcineurin in mediating adaptations in myosin heavy chain (MHC) isoform expression and muscle mass using 3-month old wild-type (WT) and transgenic mice displaying high-level expression of a constitutively active form of calcineurin (MCK-CN* mice).

**Results:**

Slow muscles, e.g., soleus, were significantly larger (by ~24%), whereas fast muscles, e.g., medial gastrocnemius (MG) and tibialis anterior were significantly smaller (by ~26 and ~16%, respectively) in MCK-CN* mice compared to WT. The masses of mixed phenotype muscles, such as the plantaris and the extensor digitorum longus, were not significantly changed from WT. The soleus, plantaris, MG and diaphragm displayed shifts toward slower MHC isoforms, e.g., soleus from WT mice contained ~52% MHC-I, ~39% MHC-IIa, and ~9% MHC-IIx, whereas MCK-CN* mice had ~67% MHC-I, ~26% MHC-IIa, and ~7% MHC-IIx. The specific isoforms that were either up or down-regulated were muscle-specific. For instance, the proportion of MHC-IIa was decreased in the soleus and diaphragm, but increased in the plantaris and MG of MCK-CN* mice. Also, the proportion of MHC-IIx was unchanged in the soleus, decreased in the diaphragm and increased in the plantaris and MG of MCK-CN* relative to WT mice. Fast to slow shifts in fiber type proportions were evident for the plantaris, but not the soleus. Fast, but not slow, plantaris fibers of MCK-CN* mice had higher oxidative and lower glycolytic properties than WT.

**Conclusion:**

These data suggest that calcineurin activation can influence muscle phenotype and that the specific influence of calcineurin activation on the phenotypic and mass characteristics of a muscle is dependent upon the original phenotypic state of the muscle.

## Background

Skeletal muscle adapts to neural and use-dependent signals by altering its mass and phenotypic properties, including the proportion of slow (I) and fast (IIa, IIx, and IIb) myosin heavy chain (MHC) isoforms [1,2]. Calcineurin has been implicated as a regulatory molecule involved in the transduction of contractile activity-based signals to molecular signals involved in the regulation of muscle growth and phenotypic gene expression in skeletal muscle [3-14]. Calcineurin is a phosphatase (protein phosphatase 2B) that is activated by calmodulin upon binding Ca^2+ ^and has been shown to play an important role in the regulation of cytokine gene expression in lymphocytes [15,16]. Substrates for calcineurin include the phosphorylated isoforms of a transcription factor known as the nuclear factor of activated T cells (NFAT) [17]. Upon dephosphorylation, NFAT isoforms translocate into the nucleus where they can bind to a conserved DNA binding site known as the NFAT response element (NRE) and alter transcription, leading to enhanced expression of specific genes [15-17].

It has been proposed that in skeletal muscle nuclear NFAT can activate the expression of slow muscle phenotypic genes [3-5,8,11,14]. In addition, MEF2 and NFAT appear to act synergistically in turning on slow muscle fiber specific genes [18]. Thus, increased muscle contractile activity and the resulting sustained elevations in cytosolic Ca^2+ ^could potentially result in altered phenotype-specific gene expression via calcineurin [4]. To date, however, no studies have reported the influence of chronically elevated calcineurin activity on the MHC isoform composition and fiber cross-sectional areas (CSAs) of limb and respiratory skeletal muscles. Therefore, in the present study, transgenic mice containing a highly expressed transgene consisting of a constitutively active form of calcineurin (CN*) driven by the muscle creatine kinase (MCK) enhancer were used to assess the influence of chronic calcineurin activation on MHC isoform protein and muscle mass and fiber cross-sectional area (CSA). Previous studies using the same line of transgenic mice demonstrated that calcineurin activation elevates the expression of some slow phenotypic genes [11], the percentage of EDL fibers with MHC-IIa [19], and proteins related to insulin-stimulated glucose uptake [20].

## Results

**Figure 1:**
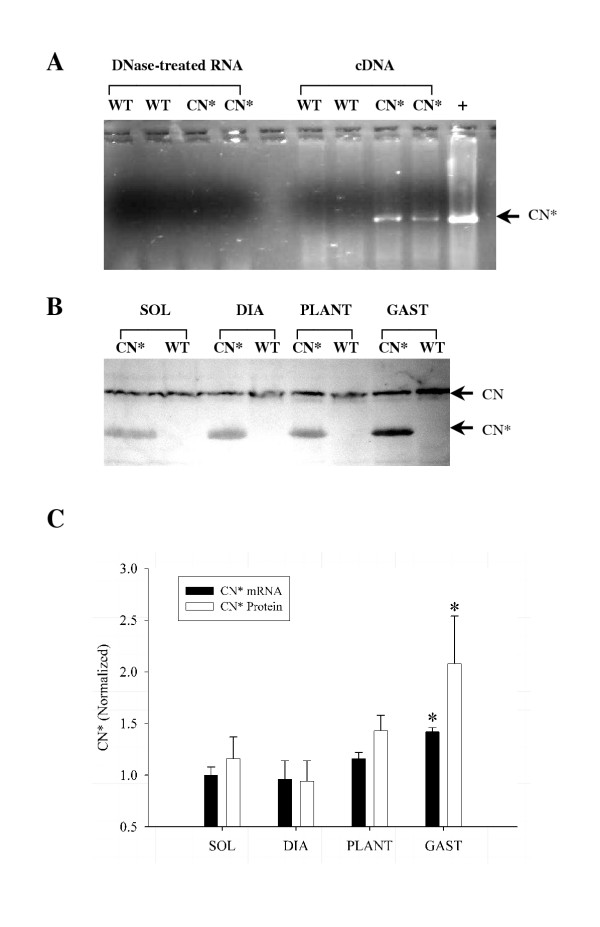
A) SYBR^® ^Green I stained agarose gel of PCR products following RT-PCR for the MCK-CN* mRNA product. No specific product (~900 bp, arrow) is observed in the DNase-treated RNA samples (first 4 lanes) of either wild-type (WT) or MCK-CN* transgenic (CN*) mouse gastrocnemius. Therefore, there is no contaminating genomic DNA in the RNA samples used for RT-PCR. As expected, RT-PCR of the cDNA (second four lanes) reveals that expression of the transgene is only observed in the MCK-CN* mice. A positive (+) control of tail DNA from an MCK-CN* mouse is shown on the right. B) Western blot of CN* and WT mouse soleus (SOL), diaphragm (DIA), plantaris (PLANT) and gastrocnemius (GAST) muscles for calcineurin. The polyclonal MAb recognized both the endogenous (CN, ~60 kDa) and constitutively active (CN*, ~45 kDa) forms. As expected, muscle from WT muscles did not contain the CN* protein. C) Relative semi-quantitative analysis of CN* mRNA (via RT-PCR) and protein (western blotting) in muscles of MCK-CN* mice. All values were normalized to the same soleus muscle (n = 3 – 6 per group). The asterisk (*) denotes significantly different (p ≤ 0.05) from other muscles.

The MCK-CN* transgenic mice expressed the transgene in all muscles tested as shown in Figure 1. As expected, due to transgene expression being driven by a fast muscle-specific enhancer (MCK enhancer), the fast MG muscle displayed the highest level of expression. The soleus (a mixed fast and slow muscle in mice), diaphragm and plantaris showed relatively similar levels of transgene expression.

**Figure 2:**
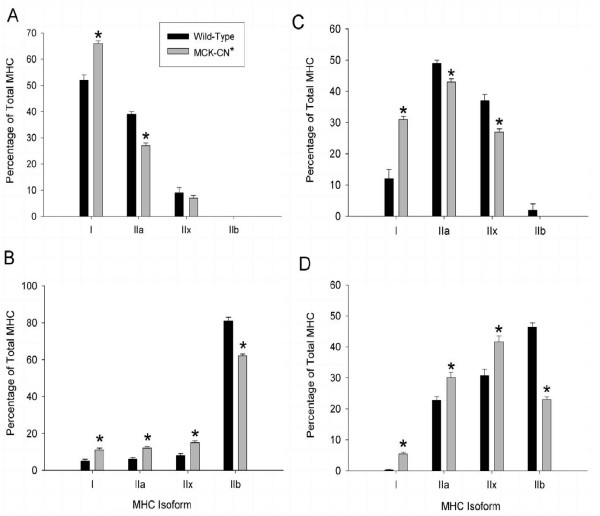
Adult myosin heavy chain (MHC) isoform proportions of the soleus (A), medial gastrocnemius (B), diaphragm (C), and plantaris (D) muscles of wild-type (dark bars) and MCK-CN* transgenic (gray bars) mice as determined by SDS-PAGE of whole muscle extracts. The * denotes significantly different from wild-type at p ≤ 0.05. Note the elevated proportions of slower MHC isoforms in the transgenic mice compared to wild-type.

As shown in Figure 2, the muscles from MCK-CN* mice showed significant shifts towards slower MHC isoforms compared to WT. However, the specific isoforms that were either up- or down-regulated were muscle specific. For instance, the soleus of MCK-CN* mice showed an elevation in MHC-I at the expense of MHC-IIa. The MG showed a reduction in MHC-IIb, the fastest of the MHC isoforms expressed in mice, and elevations in MHCs-I, -IIa, and -IIx. The diaphragm showed an elevation in MHC-I and reductions in MHCs-IIa and -IIx. The diaphragm of the MCK-CN* mice also showed a complete absence of MHC-IIb, which was present in small proportions in WT mice. The plantaris of MCK-CN* mice showed similar directional adaptations in MHC isoforms as did the MG, i.e., higher proportions of MHCs-I, -IIa, and -IIx, and reduced MHC-IIb relative to WT mice. Thus, in MCK-CN* mice, MHC-I was consistently elevated and MHC-IIb reduced (if it was present in the WT muscle). In contrast, MHC-IIa was increased in the MG and plantaris, but decreased in the soleus and diaphragm of MCK-CN* mice. Also, MHC-IIx was increased in the MG and plantaris, decreased in the diaphragm, and unchanged in the soleus of MCK-CN* mice relative to WT.

**Figure 3:**
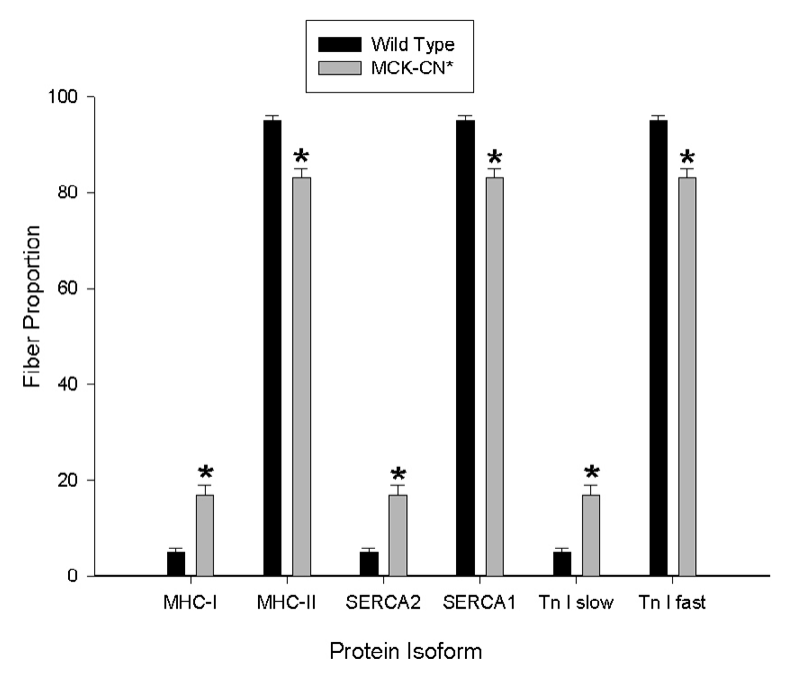
Proportions of plantaris fibers staining positively for slow and fast isoforms of MHC, sarco(endo)plasmic reticulum Ca^2+ ^ATPase (SERCA), and troponin I (Tn I) in wild-type and MCK-CN* transgenic mice. MHC-I, SERCA2, and TN I slow are the slow muscle isoforms and MHC-II, SERCA1 and Tn I fast are the fast muscle isoforms. The * denotes significantly different from wild-type at p ≤ 0.05. Note the elevated proportions of fibers staining positively for the slow isoforms in the transgenic mice compared to wild-type. All fibers that stained positively for the slow MHC (MHC-I) were positive for the slow isoforms of SERCA and Tn I and all fibers that stained positively for the fast MHC (MHC-II) were positive for the fast isoforms of SERCA and Tn I.

**Figure 4:**
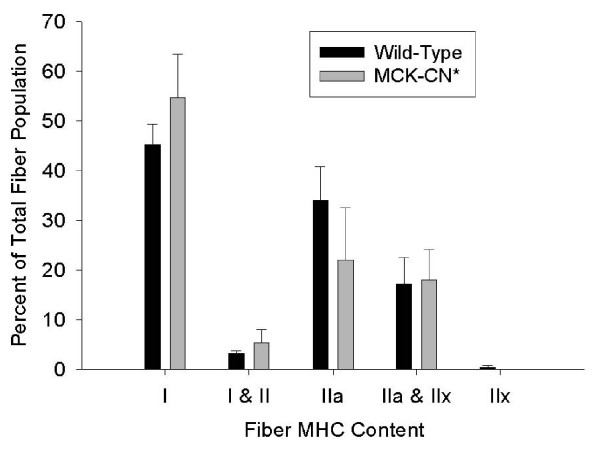
Proportions of soleus fibers staining positively for specific fast and slow MHC isoforms in wild-type and MCK* transgenic mice. No fibers stained positively for MHC-IIb with mAb BF-F3, thus antibody RT-D9 which is specific for MHC-IIx and MHC-IIb indicated the presence of MHC-IIx. No significant differences in fiber proportions of the soleus were found between wild-type and MCK-CN* mice.

The plantaris muscle of MCK-CN* mice showed a significant shift in fiber type proportions from fast to slow as measured by the proportions of fast and slow isoforms of MHC, sarco(endo)plasmic reticulum Ca^2+^-ATPase (SERCA), and troponin I (Figure 3). Plantaris fibers that stained positively for the slow isoform of one protein stained positively for the slow isoforms of the other proteins and likewise for fast protein isoforms. In contrast, immunohistochemical techniques did not detect significant differences in the proportions of fibers containing specific MHC isoforms in the soleus of MCK-CN* mice (Figure 4). However, there was a non-statistically significant tendency for increased proportions of fibers with slower MHC isoforms.

**Figure 5:**
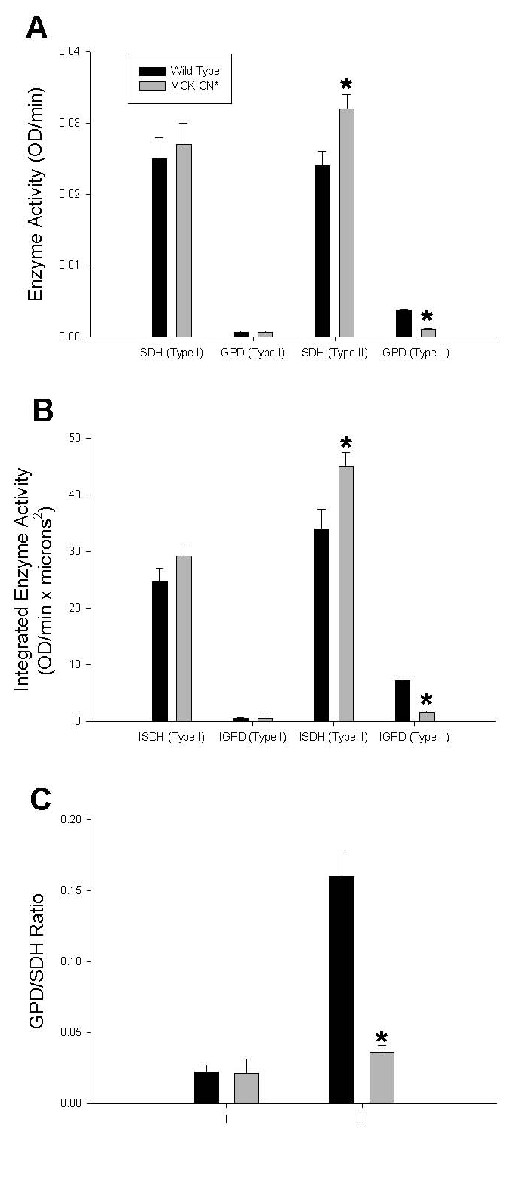
Oxidative and glycolytic enzymatic profiles of individual plantaris fibers of wild-type and MCK-CN* transgenic mice. The * denotes significantly different from wild-type at p ≤ 0.05. A) The specific activity of succinate dehydrogenase (SDH, oxidative marker) was increased in fast (type II) plantaris fibers, but unchanged in slow (type I) fibers. The specific activity of α-glycerophosphate dehydrogenase (GPD, glycolytic marker) was significantly decreased in fast plantaris fibers, but not slow fibers. B) The integrated activity (see methods for explanation of integrated activity) of succinate dehydrogenase (SDH, oxidative marker) was increased in fast (type II) plantaris fibers, but unchanged in slow (type I) fibers. The integrated activity of α-glycerophosphate dehydrogenase (GPD, glycolytic marker) was significantly decreased in fast plantaris fibers, but not slow fibers. C) The GPD/SDH ratio (glycolytic/oxidative ratio) was significantly decreased in fast fibers, but unchanged in slow fibers of the plantaris of MCK-CN* mice relative to wild-type.

To ascertain if other phenotypic properties of muscle fibers were influenced by calcineurin activation, plantaris fibers of MCK-CN* and WT mice were analyzed for marker enzymes of oxidative (succinate dehydrogenase, SDH) and glycolytic (α-glycerophosphate dehydrogenase, GPD) capacity. Fast plantaris fibers of MCK-CN* mice showed a significant increase in SDH staining intensity, indicative of elevated mitochondrial content, and a decrease in glycolytic staining intensity, indicative of reduced glycolytic capacity (Figure 5). In contrast, slow plantaris fibers of MCK-CN* mice were not different from WT (Figure 5).

As shown in Table 1, the mass of a characteristically slow muscle, i.e., the soleus, was greater; whereas, the mass of characteristically fast muscles, i.e., the MG and tibialis anterior were lower in MCK-CN* vs. WT mice. The masses of muscles that characteristically display a more intermediate phenotype, with relatively high proportions of fast oxidative (i.e., IIa and IIx) fibers, i.e., the plantaris and the extensor digitorum longus, were unchanged.

**Table 1:** Body and absolute and relative muscle masses.

	Wild-type (n = 6)	MCK-CN* (n = 10)
Body Mass (g)	23.3 ± 0.9	20.7 ± 0.4*
Absolute Muscle Mass (mg)		
Soleus	8.2 ± 0.6	10.2 ± 0.6*
MG	46.7 ± 1.8	34.5 ± 1.9*
PLT	16.8 ± 0.8	16.2 ± 0.7
TA	43.0 ± 1.2	36.0 ± 1.3*
EDL	8.3 ± 1.3	7.8 ± 0.5
Relative Muscle Mass (mg/g body mass)		
Soleus	0.35 ± 0.02	0.48 ± 0.02*
MG	2.01 ± 0.04	1.66 ± 0.09*
PLT	0.72 ± 0.03	0.78 ± 0.04
TA	1.85 ± 0.04	1.74 ± 0.06
EDL	0.36 ± 0.05	0.38 ± 0.03

Abbreviations: MG, medial gastrocnemius; PLT, plantaris; TA, tibialis anterior; EDL, extensor digitorum longus. Values with asterisks are significantly different from wild-type at p ≤ 0.05.

**Figure 6:**
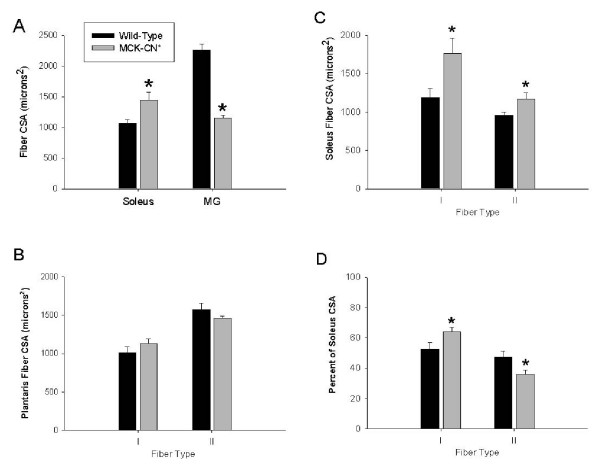
Individual fiber cross sectional areas (CSAs) in muscles of wild-type and MCK-CN* mice. A) Mean fiber CSAs in soleus and medial gastrocnemius (MG) muscle regardless of fiber type. B) Mean fiber CSAs of slow (type I) and fast (type II) fibers of the plantaris muscle. C) Mean fiber CSAs of slow (type I) and fast (type II) fibers of the soleus muscle. D) The percent of the entire soleus muscle cross-section occupied by slow (type I) and fast (type II) fibers. The * denotes significantly different from wild-type at p ≤ 0.05.

As expected, based on the overall differences in muscle mass between MCK-CN* and WT mice, the soleus contained fibers with CSAs greater than WT, whereas the MG contained fibers with CSAs smaller than WT (Figure 6A). The plantaris, which showed no change in mass, also showed no change in fiber CSA (Figure 6B). In the soleus, both slow and fast fibers were larger (Figure 6C). However, slow fibers were increased in CSA by 49% and fast fibers by only 22%. This resulted in a ~20% increase in the proportion of the muscle CSA occupied by slow fibers in MCK-CN* mice (Figure 6D). Thus, the changes in overall phenotype of the soleus can be partially accounted for by changes in the size of specific types of fibers.

## Discussion

Elevated calcineurin activity altered the phenotypic profile and mass of both fast and slow muscles and muscle fibers. The phenotypic changes were indicated by the general fast to slow adaptation in MHC isoform expression in MCK-CN* versus WT mice. The changes in phenotype were apparently not restricted to MHC isoforms since the plantaris of MCK-CN* mice had higher proportions of fibers that stained positive for the slow isoforms of MHC, SERCA and troponin I, suggesting a coordinated increase in the expression of slow phenotypic proteins upon calcineurin activation. Fast plantaris fibers also had elevated oxidative and reduced glycolytic profiles, suggesting that calcineurin activation influenced the metabolic profile of fibers promoting a more oxidative phenotype. These data suggest that fibers that did not show adaptations in MHC, SERCA or troponin I isoforms and remained fast were still influenced by calcineurin activation to acquire an oxidative phenotype. These changes are consistent with the proposed role of calcineurin as a sensor of contractile activity and transducer of contractile signals into molecular signals that induce the expression of a slow oxidative phenotype [4].

The overall changes in MHC isoform composition for some muscles, such as the soleus, were apparently aided by fiber type-specific changes in the CSA of the fibers, such that type I fibers were increased and type II fibers decreased (or increased only slightly in the soleus) in CSA as a result of elevated calcineurin activity. These data are consistent with the hypothesis that elevated calcineurin activity induces increased expression of slow muscle phenotypic genes resulting in elevated mass of slow muscles and muscle fibers. In addition, fibers from fast muscles may attain a smaller CSA in MCK-CN* mice relative to WT as a result of the change in phenotype from a fast glycolytic type of fiber (typically the largest fibers) towards a slow or fast oxidative type of fiber (typically the smallest fibers), see Rivero et al. [21,22].

Although the general adaptations in phenotype were in the direction of faster to slower isoforms, the actual transitions in MHC isoform content were muscle-specific. This is reminiscent of the muscle-specific influence of thyroid hormone treatment on muscle phenotype [23]. Of the muscles examined, the diaphragm of MCK-CN* mice showed the greatest absolute elevation in MHC-I content. Also, in the soleus and diaphragm of MCK-CN* mice, MHC-I was up-regulated at the expense of MHC-IIa. However, in the MG, MHCs-I, -IIa, and -IIx were up-regulated at the expense of MHC-IIb. The mechanisms by which some muscles (MG and plantaris) respond to calcineurin activation by up-regulating MHC-IIa and IIx whereas others (soleus and diaphragm) down-regulate these MHCs is currently unknown. *In vitro *data suggest that MHC-IIa is highly (100 fold) and MHC-IIx and -IIb moderately (~5 – 10 fold) stimulated by calcineurin activation in mouse C_2_C_12 _cells [24]. Because the adaptations were muscle-specific it is likely that additional mechanisms must be invoked in the *in vivo *state to modulate the ultimate response to calcineurin activation. Since the diaphragm showed the greatest response to calcineurin activation and the diaphragm is a chronically active muscle it is tempting to speculate that the chronic motor activity of this muscle results in an up-regulation of parallel signaling pathways that may support a more pronounced calcineurin activity-induced shift in fiber phenotype.

The observation that MHC-IIa and MHC-IIx were up-regulated in fast muscles and MHC-I was up-regulated in slow muscles of MCK-CN* mice is in agreement with the idea of a limited adaptive range for muscles of varying phenotype. The limited adaptive range theory essentially states that muscles from a slow developmental lineage have the capacity to adapt in the range of I ↔ IIa ↔ IIx, whereas muscles from a fast developmental lineage can adapt in the range of IIa ↔ IIx ↔ IIb [25]. Thus, MG and plantaris fibers may be restricted to express MHC-IIa as the 'slowest' MHC isoform. In contrast, soleus and diaphragm fibers have the capacity to express MHC-I as the slowest MHC. However, recent data demonstrate that the suggested limited adaptive range for slow muscle is not strict. For example, combined treatment of rats with thyroid hormone and hindlimb suspension results in substantial expression of MHC-IIb by the soleus [26].

Another potential explanation for the muscle-specific response to chronic calcineurin activation is a possible obligatory step-wise transition from MHC-IIb ↔ IIx ↔ IIa ↔ I [27]. Following this scheme a fast muscle fiber would initially express MHC-IIx and then IIa as it transitioned towards a slower phenotype. However, this is a temporal scheme and the animals in this study were 3 months of age, therefore, sufficient time was available for a complete transition of fast muscles to MHC-I. In addition, previous studies from our [28-31] and other [26,32] laboratories have demonstrated that the obligatory step-wise transition scheme is not always followed. For instance, some rat soleus fibers can transition from MHC-I to MHC-IIx without apparent expression of MHC-IIa protein following hindlimb suspension, spaceflight, or paralysis induced by either spinal cord transection or spinal cord isolation [28-31].

The molecular mechanisms involved in directing the muscle-specific response are unknown, but could involve parallel signaling pathways, perhaps involving CAMKinase, MEF2, PGC-1, or altered histone acetylation at specific sites. It is also possible that variability in inherent motor-neuron activity influences the expression of specific sets of parallel signaling pathways which in turn allows for alteration of only specific sets of phenotypic proteins in muscles of differing phenotype. Our data showing that the diaphragm, a chronically active muscle, had the greatest absolute increase in MHC-I protein lends support to this idea.

Ultimately, these data support the idea that calcineurin activity plays a role in the regulation of muscle phenotype. Since the expression level of the transgene in these mice results in basal calcineurin activity levels that are ~10 times the activity in WT mice [33], and since the phenotypic adaptations were somewhat modest, this suggests that parallel pathways likely need to be invoked to induce a more complete fiber type shift. In addition, the activation of calcineurin did not completely supersede the developmental-based phenotypic lineage. For instance, in the MCK-CN* mice the soleus retained ~40% fast (type II) fibers and the MG was predominantly composed of fast MHC isoforms. However, it must be noted that the transgene is active in the MCK-CN* mice during all stages of development. Thus, negative feedback mechanisms that may reduce the down-stream effectiveness of calcineurin activation may be enhanced or invoked during the development and maturation of the skeletal musculature. It is also possible that parallel pathways must be invoked with calcineurin to fully activate the slow muscle specific phenotype, as suggested by Dunn et al., [7] and demonstrated by Wu et al., [18]. Presence of the transgene will only directly influence the basal calcineurin activity and it is possible that higher sustained levels of calcineurin activity, such as may occur with sustained contractile activity-induced elevations in myoplasmic calcium, would support a more complete fiber transformation. Since the Ca^2+^-calmodulin stimulation of calcineurin can be as great as 100-fold [15], it is possible that normal *in vivo *calcium-induced activation of endogenous calcineurin would result in a much greater elevation in calcineurin activity and a greater degree of fiber type transformation.

Several studies have suggested that calcineurin activation does not influence muscle mass [7,34-36] or phenotype [37]. One of these studies, Dunn et al., [7], made use of a similar transgenic mouse model as that used in this study. However, the level of basal calcineurin activity in the mice used by Dunn et al., [7] was reported to be several times lower (~same as WT) than that reported for the mice used in our study, ~10× greater than WT, i.e., transgenic mouse line #1 from reference [33]. Thus, the lack of effect in some of the previous studies may be related to the level of transgene expression and the lower basal calcineurin activity. It is interesting to note that the transgenic mice used by Dunn et al. [7], also did not display differences in fiber phenotype compared to WT. In contrast, other studies have demonstrated that calcineurin activation and or the accumulation of nuclear NFAT may be involved in the mediation of growth inducing factors, such as IGF-1 [10,13,38].

Curiously, our data suggest that chronic calcineurin activation results in elevated mass of slow muscle and reduced mass of fast muscle. This suggests that the changes in mass and fiber CSA may be secondary to the changes in phenotype. The differential effect of chronic calcineurin activation on slow vs. fast muscle mass may be explained as follows. Elevated expression of slow phenotypic genes in a slow muscle could theoretically result in an increased accretion of slow phenotypic proteins and elevated size of slow fibers. Fast muscle fibers showed an elevation in oxidative enzyme activity, i.e., transitioned from a fast glycolytic (FG) to a fast oxidative glycolytic (FOG) phenotype. Typically, FG fibers are larger than FOG fibers in mammalian muscle. A strong inverse correlation exists between oxidative capacity and fiber CSA [39,40]. Thus, as fast fibers transitioned from FG towards FOG in response to calcineurin activation, they would achieve a smaller CSA.

## Conclusions

Collectively, the data support the hypothesis that calcineurin activation plays an important role in the modulation of skeletal muscle phenotype. The responses to calcineurin activation were muscle specific, suggesting that inherent differences in signaling mechanisms within distinct muscles likely modulate the response to calcineurin activation. The observation that a 10-fold increase in basal calcineurin activity results in alterations in the expression of fast versus slow phenotypic proteins and alterations in the metabolic capacities of fibers that do not show a transition to slow fiber type isoforms suggests that the activation of this pathway can modulate multiple aspects of fiber phenotype. However, parallel pathways likely need to be invoked to completely alter the phenotype of all fibers in a muscle.

## Methods

### Animals

Breeding pairs of MCK-CN* transgenic mice were obtained from the primary colony at the University of Texas Southwestern Medical Center [11] and housed at the Animal Care Facility at Virginia Polytechnic Institute and State University. This specific line of transgenic mice has been used previously to study the influence of calcineurin activation on the expression of utrophin [19], and insulin-sensitive glucose uptake [20]. Female B6C3F1 mice expressing a transgene consisting of 4800 bp of the MCK enhancer, the coding sequence of the activated form of calcineurin, which codes for amino acids 1 to 398 and lacks the autoinhibitory and calmodulin binding domains [41], and 620 bp of the human growth hormone polyadenylation signal (MCK-CN* mice) were crossed with the C57Bl6J strain. Transgenic offspring were identified by PCR amplification of tail DNA using primers specific for sequences within the calcineurin coding region (5'-GAA CCA GCA GTT CCT GTG TGT ACA CG-3') and the hGH polyadenylation signal (5'-CAC TCC AGC TTG GTT CCC GAA TAG AC-3'). Since the transgenic mice were generated in the B6C3F1 strain and were then crossed with the C57Bl6J line, all comparisons were made to wild-type (WT) littermate controls, which would share any strain dependent variables with the MCK-CN* transgenic mice. All animal procedures were approved by the Virginia Polytechnic Institute and State University and California State Polytechnic University Institutional Animal Care and Use Committees and conform to the American Physiological Society's guiding principles for the care and use of animals.

MCK-CN* (n = 10) and WT littermate controls (n = 6) were sacrificed by pentobarbital injection at 3 months of age. Animals from two independent litters were used. The diaphragm, soleus, medial gastrocnemius (MG), plantaris, tibialis anterior, and extensor digitorum longus muscles were dissected free, trimmed of excess connective tissue, and weighed (diaphragm weights were not obtained). The muscles (except tibialis anterior and extensor digitorum longus) were then rapidly frozen in melting isopentane cooled with liquid nitrogen and stored at -70°C.

### Muscle RNA analyses

Total RNA was isolated from frozen muscle (soleus, MG, plantaris, and diaphragm) samples using Trizol reagent according to the manufacturer's instructions (Invitrogen Corp., Carlsbad, CA, USA). The final RNA pellet was dissolved in nuclease-free water, treated with DNA-free™ (Ambion Inc, Austin, TX, USA) to remove potential contaminating genomic DNA, and then quantified by UV (A_260_) light absorbance. All RNA samples had A_260_:A_280 _ratios of greater than 1.8. Two μg of the isolated RNA were reverse transcribed to cDNA with Superscript II (Invitrogen Corp.) in a final volume of 20 μl. The same PCR primers used for genotyping were used to amplify the specific cDNA fragment from transgenic mice expressing the MCK-CN* transgene. The PCR reaction included: 1 μl of cDNA, 1X Platinum Taq PCR buffer (Invitrogen Corp.), 1.5 mM MgCl_2_, 0.2 mM dNTPs, 1 μM each primer, and 1 unit Platinum Taq (Invitrogen Corp.) in a final volume of 25 μl. The PCR cycle conditions were 94°C for 4 min and then 40 cycles of 94°C for 30 sec, 61°C for 30 sec, and 72°C for 30 sec, followed by a final step at 72°C for 5 min. The PCR products were separated on 1.5% agarose gels, stained with SYBR^® ^green I (Molecular Probes Inc., Eugene, OR, USA) and quantified using a ChemiImager 5500 (Alpha Innotech, San Leandro, CA, USA).

### Muscle protein analyses

One MG, plantaris and soleus muscle from each animal were serially sectioned perpendicular to the fiber axis using a Microm cryostat, stained imuunohistochemically for fast or slow myosin heavy chain (MHC) isoforms using either anti-fast or anti-slow MHC monoclonal antibodies (mAb, Novocastra Laboratories, Inc.) according to Talmadge et al., [30]. Soleus tissue sections were also analyzed for the presence of MHC-IIa, MHC-IIx and MHC-IIb using mAbs specific for MHC-IIa (mAb SC-71), MHCs-IIx & -IIb (mAb RT-D9) and MHC-IIb (mAb BF-F3). Serial sections of the plantaris were also immunohistochemically stained for the presence of fast and slow isoforms of troponin I (Research Diagnostics Inc.) and sarco(endo)plasmic reticulum Ca^2+ ^ATPase (SERCA, Novocastra Laboratories, Inc.) isoforms using similar techniques. The sections treated for immunohistochemistry were used to measure the cross-sectional area (CSA) of fifty individual muscle fibers per muscle using a calibrated microscopy-based image analysis system consisting of a GMS-300 grayscale microscopy system (Scion, Frederick, MD) and a Nikon Eclipse E400 microscope.

The MG, plantaris and soleus from the opposite limb and the whole diaphragm (including costal and crural portions) were subjected to high resolution SDS-PAGE for analysis of myosin heavy chain isoforms as detailed in Talmadge and Roy [42]. These muscles were also analyzed for constitutively active and endogenous calcineurin protein levels by semi-quantitative western blotting as described in detail previously by Spangenburg et al. [43]. The primary antibody used for western blotting was a rabbit anti-calcineurin Pan A polyclonal (AB1695) from Chemicon International (Temecula, CA, USA) which recognized both the constitutively active (CN*) and endogenous forms of calcineurin. The western blot results obtained using this primary antibody were verified (data not shown) with other antibodies that showed similar patterns of reactivity, including antibodies CN-A1 (Sigma Chemical Co., St. Louis, Mo, USA), SC-9070 (Santa Cruz Biotechnology Inc., Santa Cruz, CA, USA), and AB1696 (Chemicon International).

### Quantitative histochemistry of individual muscle fibers

Quantitative histochemical analyses of oxidative and glycolytic enzyme activities were measured on 50 plantaris fibers per muscle that were immunohistochemically typed for fast or slow MHC isoform content in serial sections. According to Rivero et al., [21,22], succinate dehydrogenase (SDH; EC 1.3.5.1) was used as a marker for oxidative capacity and α-glycerophosphate dehydrogenase (GPD) was used as a marker for glycolytic capacity of individual muscle fibers. Histochemical staining and image analysis were performed as previously described in detail [21,22]. The specific enzymatic activity per unit fiber volume (i.e., enzyme concentration) is expressed as change in OD per minute of reaction. The integrated enzymatic activities (ISDH and IGPD) were calculated as the product of specific enzymatic activity (OD/min for a given fiber) and CSA of that fiber which reflects the enzyme content per fiber and is expressed as OD/min × microns^2^.

### Statistical analyses

Student's t-tests were used for comparing mean data between transgenic and WT mice at each age. Statistical differences were significant at p ≤ 0.05.

## Authors' contributions

RJT: conception, design, data collection and analysis, manuscript preparation, research funds collection.

JSO: quantitative histochemical analyses, genotyping, statistical analysis, figure preparation.

MRR: electrophoretic analyses, statistical analysis, figure preparation.

NDG: mRNA and electrophoretic analyses, figure preparation.

SRS: western blotting, figure preparation.

SJL: muscle sample collection.

FJN: supplied transgenic mice, provided genotyping expertise.
